# A Design of FMCW Fuze System and Ranging Algorithm Based on Frequency–Phase Composite Modulation Using Chaotic Codes

**DOI:** 10.3390/s26051434

**Published:** 2026-02-25

**Authors:** Jincheng Zhang, Xinhong Hao, Chaowen Hou, Jianqiu Wang

**Affiliations:** Science and Technology on Electromechanical Dynamic Control Laboratory, Beijing Institute of Technology, Beijing 100081, China; 3120240267@bit.edu.cn (J.Z.);

**Keywords:** chaotic code, radio fuze, ambiguity function, linear frequency modulation, anti-intercept ability, chaotic bi-phased code modulation

## Abstract

To address the vulnerability of traditional linear frequency-modulated continuous wave (FMCW) fuze to jamming due to fixed modulation parameters, this paper proposes a novel fuze waveform design scheme using chaotic code-based frequency and phase composite modulation along with a Normalized Rate-Invariant Ranging algorithm (NRIR). Leveraging the ergodicity and initial value sensitivity of the Logistic chaotic map, a dual-dimensional composite modulation system is constructed. In the frequency domain, the frequency modulation slope undergoes periodic binary variation according to chaotic states to break the signal periodicity. In the phase domain, phase encoding is implemented based on chaotic binary sequences to further improve waveform entropy and complexity, effectively destabilizing the parameter stability required for coherent jamming. To resolve the distance–Doppler coupling challenges and spectral dispersion issues caused by variable-slope modulation, the NRIR algorithm is developed. By introducing a resampling transformation operator, the non-stationary rate-varying beat frequency signal is mapped to a normalized “constant-slope” space, enabling coherent accumulation and ranging of targets. Using the ambiguity function as an analytical tool, theoretical analyses, simulation experiments, and test results demonstrate that this design scheme exhibits excellent performance in suppressing DRFM jamming and sweep-frequency jamming, providing theoretical support and technical approaches for fuze anti-jamming design.

## 1. Introduction

The frequency-modulated continuous wave fuze offers distinct advantages, including high range resolution, low peak power requirements, and cost-effectiveness. These attributes have led to widespread application in various conventional munitions such as grenades, mortar shells, and missiles [1]. However, with the rapid advancement of electronic warfare (EW) technologies, the large-scale deployment of digital jamming equipment poses a severe challenge to the anti-jamming performance of fuzes in modern battlefield environments [2]. Among these threats, active deception jamming equipment centered on Digital Radio Frequency Memory (DRFM) technology, alongside sweep-frequency jamming characterized by wideband and high-power coverage, constitutes the most significant threat to radio fuzes [3]. To enhance battlefield survivability of fuzes, extensive research has been conducted, primarily focusing on two fronts: waveform design and anti-jamming signal processing algorithms.

Chaos is a deterministic, nonlinear dynamical phenomenon characterized by quasi-randomness, extreme sensitivity to conditions and the presence of strange attractors. Due to these properties, chaotic signal has been widely applied in fields like secure communications [4]. In recent years, the application of chaos in radar and sensors has gained traction. For instance, Pappu et al. [5] proposed an integrated sensing and communication system based on multiplexed chaotic waveforms, verifying the robustness of chaotic signals in multipath and interference environments. Similarly, Wu et al. [6] designed an anti-interference ultra-wideband (UWB) fuze waveform based on chaotic Pulse Position Modulation (PPM), which effectively reduced the probability of signal interception. Additionally, Li et al. [7] proposed a radar waveform design method based on multicarrier phase coding for suppressing autocorrelation sidelobes, demonstrating the potential of chaotic coding in optimizing radar performance.

Regarding specific anti-jamming techniques, Han et al. [8] proposed a DRFM reconstruction and cancellation method based on accurate edge detection, which estimates jamming parameters through time-domain deconvolution to achieve cancellation. However, this method entails high computational complexity and lacks the real-time performance required for expendable fuze applications. Yazar et al. [9] introduced a frequency-shifted FMCW method that utilizes Voltage Controlled Oscillators (VCOs) in the analog domain to frequency-shift sub-pulses to counter DRFM jamming. Nevertheless, most current research applying chaotic sequences to FMCW fuzes focuses on phase modulation. While this approach increases waveform complexity, demodulation in strong jamming environments remains complex, and it often leads to elevated sidelobes that affect range resolution [10]. Furthermore, against sweep-frequency jamming, non-coherent jamming often results in a high false alarm rate [11].

In summary, existing research exhibits the following limitations:

1. Singular Modulation Type: Modulation is largely concentrated on phase modulation, which is susceptible to parameter interception by DRFM jammers that can obtain pulse compression gain through delayed forwarding.
2. Restricted Range Resolution: Although variable parameter signals (such as variable slope) can theoretically defeat jamming, processing such different frequency signals prevents direct multi-period coherent accumulation. This results in spectral dispersion that severely impacts range resolution and SJR improvement.
3. High Real-Time Complexity: Methods based on deep learning or complex iterative reconstruction are difficult to implement in real time on resource-limited fuze platforms.

To address these issues, this paper proposes a composite modulation system based on chaotic code frequency slope and phase. We design a Normalized Rate-Invariant Ranging (NRIR) algorithm that achieves coherent focusing of variable-rate signals through mathematical transformation. By deriving the ambiguity function for this composite modulation signal, we demonstrate that it possesses ideal range and velocity resolution, effectively balancing anti-jamming performance with engineering feasibility.

## 2. Chaotic Dynamics and Parameter Mapping Model

### 2.1. Logistic Mapping and Properties

The system employs the Logistic map for chaotic sequence generation, which is defined as follows:

(1) $$x_{n+1}=\mu x_{n}(1-x_{n})$$

where $x_{n}\in (0,1)$ is the *n*-th state variable, and μ=4 ensures a fully chaotic state.

The sequence exhibits ergodicity, following the Chebyshev density [12]:

(2) $$\rho (x)=\frac{1}{\pi \sqrt{x(1-x)}}$$

**Figure 1:**
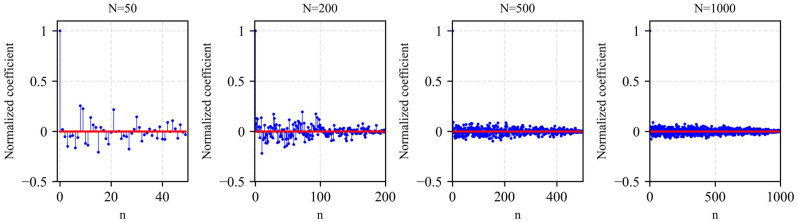
Logistic sequence autocorrelation performance image.

Furthermore, the sequence has high sensitivity to initial values and excellent autocorrelation, ensuring unpredictability against interceptors. The image of autocorrelation of Logistic sequence varying with sequence length is shown in Figure 1.

### 2.2. Phase Modulation Properties

The modulation parameters are mapped from the chaotic state $\left\{x_{n}\right\}$ to ensure waveform agility. Phase Modulation: A binary sequence $\left\{c_{k,n}\right\}$ is generated via thresholding:

(3) $$c_{k,n}=\left\{\begin{array}{ll} -1, & \;x_{n}\leq x_{\mathrm{aver}}\\ 1, & \;x_{n}>x_{\mathrm{aver}} \end{array}\right.$$

where $x_{\mathrm{aver}}$ is the mean value of the Logistic sequence.

Slope Modulation: The chirp period $\left\{T_{p,n}\right\}$ varies linearly with $\left\{x_{n}\right\}$:

(4) $$T_{p,n}=T_{\min}+c_{k,n}(T_{\max}-T_{\min})$$

Maintaining a constant sweep bandwidth *B*, the frequency modulation slope K for the *n*-th period is expressed as

(5) $$K_{n}=\frac{B}{T_{p,n}}=\frac{B}{T_{\min}+c_{k,n}(T_{\max}-T_{\min})}$$

This ensures that $K_{n}$ jumps chaotically between periods, preventing the jammer from locking onto a stable chirp rate.

Regarding phase modulation, the chaotic state is used to generate the binary sequence $\left\{c_{k,n}\right\}$, which generates symbols through threshold decision for Binary Phase Shift Keying (BPSK). Let the symbol width be $\tau_{c}$. The number of symbols within each modulation period $N_{c}=\left\lfloor T_{p,n}/\tau_{c}\right\rfloor$ varies chaotically with the period.

## 3. Composite Modulation Signal Model

### 3.1. Transmitted and Received Signals

The transmitted signal consists of a train of pulses with variable slopes and chaotic phase codes. The start time of the n-th pulse $t_{n}$ is

(6) $$t_{n}=\sum_{i=0}^{n-1}T_{p,i}.$$

The phase code function is

(7) $$p_{n}(t)=\sum_{k=0}^{N_{c}-1}c_{k,n}\cdot \mathrm{rect}((t-k\tau_{c})/\tau_{c})$$

For $t\in [t_{n},t_{n}+T_{p,n})$, the complex envelope of the transmitted signal is expressed as follows:

(8) $$s_{tx,n}(t)=A_{0}p_{n}(t-t_{n})\exp\left(j\left(2\pi f_{0}t+\pi K_{n}{\left(t-t_{n}\right)}^{2}\right)\right)$$

where $A_{0}$ is the amplitude of the transmitted signal; $f_{0}$ is the carrier frequency.

This formula indicates that within each symbol interval, the transmitted signal not only has the frequency modulation characteristic but also exhibits a phase jump of 0 or *π* determined by $\left\{c_{k,n}\right\}$. To ensure the phase reference for subsequent ranging, the inter-period phase is kept continuous through phase alignment.

The total transmitted signal is the superposition of all periodic signals:

(9) $$s_{tx}(t)=\sum_{n=0}^{+\infty }s_{tx,n}\left(t\right)\cdot \mathrm{rect}\left(\frac{t-t_{n}-T_{p,n}/2}{T_{p,n}}\right)$$

Assume that the target is a point target with distance *R* and radial velocity *v*. Then the time delay $\tau_{t}$ is expressed as

(10) $$\tau_{t}=\frac{2(R-vt)}{c}=\tau_{0}-\frac{2v}{c}\cdot t$$

where $\tau_{0}$ is the time delay at the reference moment.

The echo signal is a delayed version of the transmitted signal [12]. Within the *n*-th processing period, since the speed of light *c* is much greater than the target velocity *v* and the symbol width is relatively short, the time delay can be approximately regarded as a constant within a single symbol period. Under this condition, the target echo for the *n*-th period can be expressed as

(11) $$s_{rx,n}(t)=A_{0}p_{n}(t-t_{n}-\tau )\cdot \exp\left(j\left(2\pi f_{0}(t-\tau )+\pi K_{n}{\left(t-t_{n}-\tau \right)}^{2}\right)\right)$$

### 3.2. Beat Signal

The phase difference of the beat frequency signal is

(12) $$\Delta \Phi (t)=[2\pi f_{0}(t-\tau )-2\pi f_{0}t]+[\pi K_{n}{\left(t-t_{n}-\tau \right)}^{2}-\pi K_{n}{\left(t-t_{n}\right)}^{2}]$$

The local reference signal $s_{\mathrm{Ref},n}(t)$ contains the chaotic code synchronized with the transmission and the frequency modulation phase:

(13) $$s_{\mathrm{Ref},n}(t)=p_{n}(t-t_{n})\exp\left(j\left(2\pi f_{0}t+\pi K_{n}{\left(t-t_{n}\right)}^{2}\right)\right)$$

Then the beat frequency signal can be expressed as

(14) $$y_{n}(t)=p_{n}(t-t_{n})p_{n}(t-t_{n}-\tau )\cdot A_{IF}\exp(j\Delta \Phi (t))$$

where $A_{IF}$ is the amplitude of $s_{\mathrm{Ref},n}(t)$.

Equation (14) yields a coherent single-frequency tone only when the code alignment condition is met: $|\tau -\tau_{\mathrm{Ref}}|<\tau_{c}$, where $\tau_{c}$ is the chip width and $\tau_{\mathrm{Ref}}=2R_{\mathrm{burst}}/c$. The chaotic nature of $p_{n}(t)$ results in a thumbtack ambiguity function, meaning that echoes outside this alignment window remain noise-like.

For the proximity fuze application, this sensitivity is utilized as a hardware range gate. The local reference signal $s_{\mathrm{Ref}}(t)$ is intentionally delayed by a fixed time $\tau_{\mathrm{Ref}}$ corresponding to the predetermined burst distance $R_{\mathrm{burst}}$. Consequently, the system remains “silent” (outputting noise) until the target enters the range gate. Once the target enters this window, the code correlation recovers the beat signal, and the NRIR algorithm is triggered to perform precise ranging within the gate. This mechanism naturally suppresses long-range clutter and ensures firing precision.

As for Equation (14), when the target echo is synchronized with the local code (τ=0), holds at this time, $p_{n}{\left(t\right)}^{2}\equiv 1$, the code modulation is completely stripped and the signal is restored to a single-frequency signal. When there is a large time delay (e.g., jamming signal), the code product term behaves as pseudo-random noise, thus achieving anti-jamming performance [13].

According to the convolution theorem, the frequency spectrum of the composite modulated signal $S_{Hyb}(f)$ is the convolution of the phase code and the frequency-modulated signal.

(15) $$S_{Hyb}(f)=\int_{-\infty }^{\infty }S_{PM}(v)S_{LFM}(f-v)dv$$

According to Wiener–Khinchin theorem,

(16) $$S_{PM}(f)=F\{(1-\frac{\left|\tau \right|}{\tau_{c}})\mathrm{rect}(\tau /\tau_{c})\}*F\{R_{c}(\tau )\}$$

(17) $$S_{LFM}(f)=\frac{1}{\sqrt{K}}rect\left(\frac{f}{B}\right)exp\left(-j\frac{\pi f^{2}}{K}\right)$$

where $R_{c}(\tau )$ is the autocorrelation function of the phase encoding signal. Since the chaotic code sequence possesses the ideal discrete autocorrelation property, we can get $R_{c}(\tau )\approx \delta (\tau )$. $\mathrm{rect}\left(f/B\right)$ indicates that the frequency spectrum is approximately constant within the range of [−B/2,B/2]. Substituting Equations (15) and (16) yields

(18) $$S_{Hyb}(f)\approx \frac{1}{\sqrt{\left|K\right|}}\int S_{PM}(\nu )e^{-j\pi {\left(f-\nu \right)}^{2}/K}rect\left(\frac{f-\nu }{B}\right)d\nu$$

**Figure 2:**
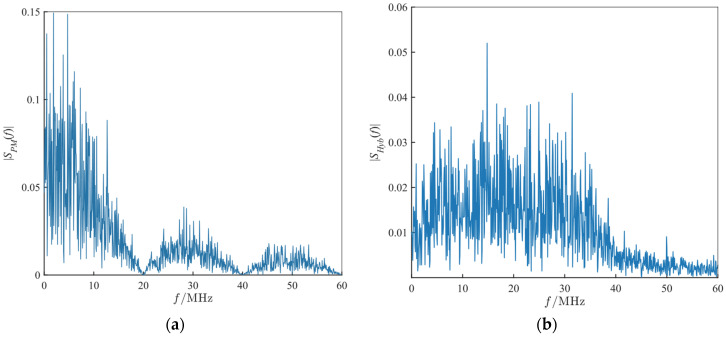
Spectra of the modulation components. (**a**) Spectrum of the chaotic phase-modulated signal $S_{PM}(f)$; (**b**) spectrum of the final frequency-phase composite modulated signal $S_{Hyb}(f)$.

It can be derived from Equation (18) that the convolution operation is equivalent to smoothing the shape of $S_{PM}(f)$ within the modulation bandwidth *B*, and the frequency spectrum is close to a rectangular shape [14,15] with the following characteristics: (1) spectrum flattening, where the rectangular spectrum characteristic of frequency modulation fills the zero points and low-energy regions of the phase code spectrum, making the energy uniformly distributed over the entire bandwidth *B*, and (2) edge steepness, determined by the bandwidth *B* rather than the symbol width. The frequency spectrums of the chaotic code phase-modulated signal and the composite modulated signal are shown in Figure 2.

### 3.3. Derivation of the Ambiguity Function

According to the two forms of the ambiguity function introduced in reference [13], the expressions of the autocorrelation ambiguity function and cross-correlation ambiguity function of the composite modulated signal can be written as Equations (19) and (20) respectively.

(19) $$\chi_{hma}(\tau ,f_{d})=\int_{-\infty }^{\infty }u(t)u^{*}(t-\tau )e^{j2\pi f_{d}t}dt$$

(20) $$\chi_{hmc}(\tau ,f_{d})=\int_{-\infty }^{\infty }u_{1}^{*}(t)u_{2}(t+\tau )e^{j2\pi f_{d}t}dt$$

where $f_{d}$ is the Doppler frequency.

(21) $$u_{1}(t)=\mathrm{rect}\left(\frac{t}{T}\right)e^{j\pi K_{1}t^{2}}$$

(22) $$u_{2}(t)=\mathrm{rect}\left(\frac{t}{T}\right)e^{j\pi K_{2}t^{2}}$$

Substituting and simplifying yields

(23) $$\chi_{hma}(\tau ,f_{d})=e^{-j\pi K\tau^{2}}\int \mathrm{rect}\left(\frac{t}{T}\right)\cdot \mathrm{rect}\left(\frac{t-\tau }{T}\right)\cdot p_{n}(t)p_{n}^{*}(t-\tau )e^{j2\pi (f_{d}+K\tau )t}dt$$

(24) $$\chi_{hmc}(\tau ,f_{d})=\int \mathrm{rect}\left(\frac{t}{T}\right)e^{-j\pi K_{1}t^{2}}\cdot \mathrm{rect}\left(\frac{t+\tau }{T}\right)e^{j\pi K_{2}{\left(t+\tau \right)}^{2}}\cdot e^{j2\pi f_{d}t}dt$$

We define $\chi_{\mathrm{code}}(\tau ,\nu )$ as

(25) $$\chi_{\mathrm{code}}(\tau ,\nu )≜\int \mathrm{rect}\left(\frac{t}{T}\right)\cdot \mathrm{rect}\left(\frac{t-\tau }{T}\right)p_{n}(t)p_{n}^{*}(t-\tau )e^{j2\pi \nu t}dt$$

Then we can get

(26) $$\chi_{hma}(\tau ,f_{d})|=|\chi_{\mathrm{code}}(\tau ,f_{d}+K\tau )|$$

The derivation of the cross-ambiguity function proceeds by evaluating the integral of the product of two LFM waveforms with mismatched slopes $K_{1}$ and $K_{2}$. Expanding the phase term yields a quadratic function of time t:

(27) $$\Phi (t)=\pi \Delta Kt^{2}+2\pi (K_{2}\tau +f_{d})t$$

By completing the square, the integral can be mapped to the standard Fresnel integrals C(u) and S(u). The integration limits $s_{a}$ and $s_{b}$ are the dimensionless time coordinates transformed into the Fresnel domain, defined as

(28) $$s_{a,b}=\sqrt{2|\Delta K|}\left(T_{1,2}+\frac{K_{2}\tau +f_{d}}{\Delta K}\right)$$

where $T_{1,2}$ define the overlapping time interval of the pulses; $\Delta K=K_{2}-K_{1}$.

Then, Equation (24) is transformed into the windowed standard Fresnel form:

(29) $$\chi_{hmc}(\tau )=e^{j\phi (\tau )}\cdot \frac{1}{\sqrt{\left|\Delta K\right|}}\cdot \frac{1}{\sqrt{2}}\left(\Delta C+j\mathrm{sgn}(\Delta K)\Delta S\right)(|\tau |<T)$$

where $\Delta C=C(\sqrt{2}s_{b})-C(\sqrt{2}s_{a})$, $\Delta S=S(\sqrt{2}s_{b})-S(\sqrt{2}s_{a})$, C(⋅), S(⋅) are the Fresnel integral: $S(x)=\int_{0}^{x}\sin(t^{2})dt$, $C(x)=\int_{0}^{x}\cos(t^{2})dt$.

**Figure 3:**
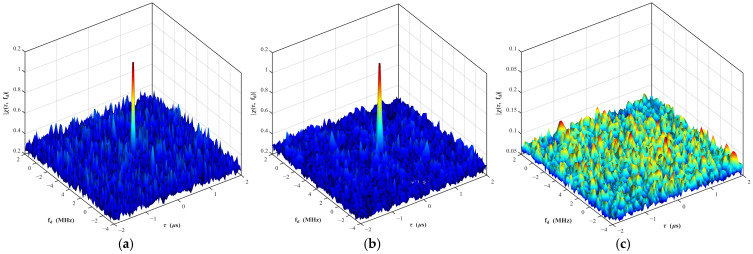
Ambiguity surfaces of two signals. (**a**) Auto-correlation ambiguity of chaotic phase-modulated signal; (**b**) auto-correlation ambiguity of hybrid-modulation signal; (**c**) cross-correlation ambiguity of hybrid modulated signal.

This normalization reveals that the correlation magnitude is scaled by the factor ΔK. Consequently, a larger slope mismatch—induced by the chaotic modulation—directly suppresses the jammer’s correlation peak, verifying the anti-jamming mechanism. The autocorrelation ambiguity diagram and cross-correlation ambiguity diagram of the composite modulated signal are shown in Figure 3.

The composite modulated waveform retains the excellent correlation characteristics of the chaotic code. The autocorrelation ambiguity function has a sharp correlation peak of thumbtack-like shape, and the value of the cross-correlation ambiguity function is very low over the entire ambiguity plane. The ambiguity diagram of the composite modulated signal is the shearing and rotation of that of the chaotic code phase-modulated signal. The sharp autocorrelation peak determines that the composite modulated signal has excellent resolution characteristics, while the low autocorrelation sidelobe and cross-correlation function determine that the composite modulated signal has extremely strong anti-performance against active deceptive jamming.

### 3.4. Interference Signal Modeling

At present, the active electronic jamming targeting frequency-modulated (FM) fuzes mainly falls into two categories: the first is barrage jamming, which degrades ranging accuracy by means of high-power and wide-bandwidth signals or noise. A representative type is sweep-frequency jamming. The second is deceptive jamming, which captures and coherently forwards fuze signals using high-speed sampling and storage technologies to generate highly deceptive false target echoes. A representative type is DRFM jamming.

#### 3.4.1. Sweep-Frequency Jamming Model

Sweep-frequency jamming achieves blanket jamming of the fuze’s operating bandwidth by varying the jamming carrier frequency regularly within a set bandwidth. Taking single-tone linear sweep-frequency jamming as an example, the sweep-frequency jamming signal can be expressed as

(30) $$x_{t}^{\mathrm{sweep}}(t)=e^{j(2\pi f_{0}t+\pi \beta_{j}{\left(t-(m-1)T_{j}\right)}^{2})}$$

where $\beta_{j}$ is the slope of the jamming signal; $T_{j}$ is the modulation period of the jamming signal.

Then, its beat frequency signal can be expressed as

(31) $$x_{j}^{\mathrm{sweep}}(t)=\sum_{m=1}^{M}e^{j\pi (\beta_{j}-\beta )t_{m}^{2}}$$

where β is the slope of the target signal; $t_{m}$ is the effective jamming moment within the corresponding jamming period.

#### 3.4.2. DRFM Jamming Model

A DRFM jammer mainly controls the signal time delay and adjusts the signal power to interfere with and suppress the real signal for frequency-modulated fuze. Its total forwarding delay $\tau_{j}$ is composed of the receiving delay $\tau_{Rj}$, processing delay $\tau_{tp}$, and the internally controlled delay c(t) of the DRFM jammer.

The actual total delay of the DRFM jammer on the received signal of the fuze can be expressed as

(32) $$\tau_{j}=2\tau_{Rj}+\tau_{tp}+c(t)=NT\pm \tau$$

The beat frequency signal can be expressed as

(33) $$x_{j}^{\mathrm{DRFM}}(t)=e^{j2\pi (f_{0}\tau_{j}+\beta \tau_{j}t-\frac{1}{2}\beta \tau_{j}^{2})}$$

**Figure 4:**
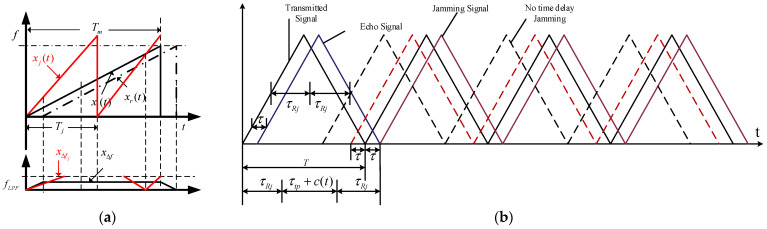
Principles of the two types of jamming. (**a**) Sweep-frequency jamming; (**b**) DRFM jamming.

DRFM jamming has strong coherence with the real target echo, and its time-domain amplitude stability is close to that of the target signal. The schematic diagrams illustrating the principles of the two types of jamming are shown in Figure 4.

## 4. Normalized Rate-Invariant Ranging Algorithm (NRIR)

Equation (13) indicates that the beat frequency signal is a single-frequency signal whose frequency is directly proportional to the target time delay. However, due to the chaotic variation in the system parameter over periods of length *n* in this paper, abrupt jumps occur within each period—even when the target distance is held constant. This renders the traditional FFT accumulation ineffective [16]. To address the spectral dispersion problem caused by the variable slope *K*, this section derives the **Normalized Rate-Invariant Ranging (NRIR)** algorithm. The core idea of this algorithm is to construct an equivalent “constant slope” signal space through time-scale transformation.

### 4.1. Description of Non-Stationarity of Rate-Varying Signals

For the *n*-th period, the beat frequency signal can be expressed as (ignoring the constant phase term)

(34) $$y_{n}(\hat{t})=\exp(-j2\pi K_{n}\tau \hat{t}),\;0\leq \hat{t}<T_{p,n}$$

where $\hat{t}=t-t_{n}$ is the fast time.

If the $y_{n}(\hat{t})$ of *N* periods is directly processed by 2D-FFT, the target energy will be dispersed over a wide frequency range of $\left[K_{\min}\tau ,K_{\max}\tau \right]$ due to the random jump of $K_{n}$ among $\left[K_{\min},K_{\max}\right]$, and thus no focused peak can be formed.

### 4.2. Derivation of the Resampling Transformation Operator

To eliminate the influence of $K_{n}$, this paper seeks a transformation T such that the transformed phase $\Phi^{\prime }({\hat{t}}^{\prime })$ is independent of $K_{n}$. First, an assumption is made according to the actual scenario: the Doppler frequency shift caused by the relative velocity between the projectile and the target is much smaller than the range beat frequency. So the Doppler term is omitted for approximation.

Define the reference slope: let $K_{\mathrm{Ref}}$ be the set reference slope (usually taking the mean value $K_{\mathrm{avg}}$ or the maximum value $K_{\max}$. Define the resampling factor:

(35) $$\alpha_{n}=\frac{K_{\mathrm{Ref}}}{K_{n}}$$

Since the receiver is aware of the current chaotic state $x_{n}$, the scalar $\alpha_{n}$ is known. Define the time-scale transformation: introduce a new time variable ${\hat{t}}^{\prime }$, such that the transformed signal $y_{n}^{\prime }({\hat{t}}^{\prime })$ satisfies the following condition:

(36) $$y_{n}^{\prime }({\hat{t}}^{\prime })=\exp\left(-j2\pi K_{\mathrm{Ref}}\tau {\hat{t}}^{\prime }\right)$$

Comparing with the phase of the original signal, we keep the phase value unchanged:

(37) $$2\pi K_{n}\tau \hat{t}=2\pi K_{\mathrm{Ref}}\tau {\hat{t}}^{\prime }$$

In digital signal processing, a signal is a discretely sampled sequence and it is necessary to obtain the signal value at the new time point $t_{k}^{\prime }=kT_{s}$. According to the above mapping, the original time point corresponding to the new time point $t_{k}^{\prime }$ is given by

(38) $${\hat{t}}_{\mathrm{req}}=\alpha_{n}t_{k}^{\prime }=\alpha_{n}kT_{s}$$

This indicates that the original sampling sequence $y_{n}[m]$ needs to be interpolated (linear interpolation) to obtain the value at time ${\hat{t}}_{\mathrm{req}}$:

(39) $$y_{n}^{\prime }[k]=\mathrm{Interp}(y_{n}[m],\hat{t}=\alpha_{n}kT_{s})$$

Since $\alpha_{n}$ varies with *n*, this constitutes a set of non-uniform resampling operations. After resampling, the signal of the *n*-th period is transformed into

(40) $$y_{n}^{\prime }(t^{\prime })=\exp\left(-j2\pi K_{n}\tau (\frac{K_{\mathrm{Ref}}}{K_{n}}t^{\prime })\right)=\exp\left(-j2\pi K_{\mathrm{Ref}}\tau t^{\prime }\right)$$

At this point, the beat frequency $f_{\mathrm{beat}}^{\prime }=K_{\mathrm{Ref}}\tau$ becomes a constant that is only related to the distance, and is independent of the transmission slope $K_{n}$. Performing the FFT transform on the resampled signal $y_{n}^{\prime }({\hat{t}}^{\prime })$ to obtain the spectral peak frequency $f_{\mathrm{peak}}$, the explicit expression for the ranging distance *R* can be derived as follows:

(41) $$R=\frac{c\cdot f_{\mathrm{peak}}}{2K_{\mathrm{Ref}}}$$

If the Doppler frequency is considered, the frequency after mapping the variable slope signal to the reference slope space through the resampling factor is

(42) $$f_{\mathrm{beat}}^{\prime }=\alpha_{n}\cdot f_{\mathrm{beat}}=\frac{K_{\mathrm{Ref}}}{K_{n}}(K_{n}\tau \pm f_{d})$$

The second item $f_{d}\cdot K_{\mathrm{Ref}}/K_{n}$ incorporates the chaotic variable $K_{n}$. This implies that the originally constant Doppler frequency shift $f_{d}$ becomes random fluctuating frequency noise after resampling. In order to demonstrate the impact of this effect, we need to quantify the size of this “Doppler jitter” relative to the FFT distance resolution unit.

Regarding the fuze parameters used in this paper, the carrier frequency $f_{0}$ is 3 GHz, the bandwidth B is 30 MHz, the pulse width $T_{p}$ is 10 μs and the maximum relative speed is 1000 m/s. We can get

(43) $$f_{d}=\frac{2v}{c}f_{0}=\frac{2\times 1000}{3\times 10^{8}}\times 3\times 10^{9}=20\;kHz$$

FFT frequency resolution is

(44) $$\Delta f_{\mathrm{FFT}}\approx \frac{1}{T_{p}}=\frac{1}{10\mu s}=100\;kHz$$

In this paper, the variation range of $K_{n}$ is 2. Then, the variation amount of the Doppler term after resampling $\Delta f_{jitter}$ is

(45) $$\Delta f_{jitter}=f_{d}\times 2=40\;kHz$$

The calculation results show that the Doppler frequency jitter introduced by the NRIR algorithm (40 kHz) is two fifths of an FFT distance unit (100 kHz). This implies that the energy is not completely dispersed and the jitter range is confined within one distance gate. The main peak still exists, slightly diffused like an interference signal. Secondly, the loss of accuracy is controllable: Although there will be a slight jump in the peak position, through multi-cycle non-coherent accumulation, this random jitter will be averaged out, thus maintaining the stability of ranging.

**Figure 5:**
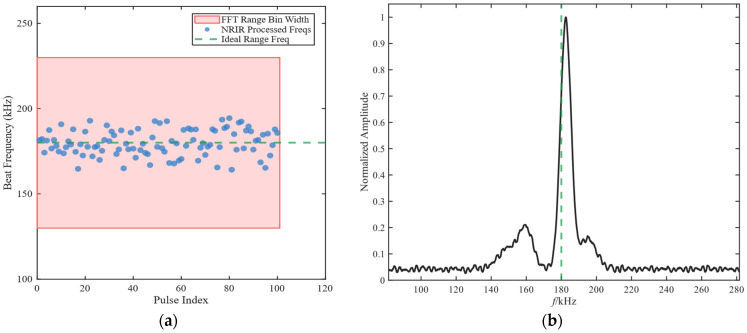
The impact of Doppler frequency on NRIR algorithm: (**a**) beat frequency jitter under Doppler frequency; (**b**) spectrum after multi-cycle non-coherent accumulation.

The simulation result is shown in Figure 5. In Figure 5a, we can clearly see that despite the high speed of 1000 m/s, the blue dots are still closely clustered within the red area. This proves that energy has not completely dissipated. In Figure 5b, although there is a fluctuation in a single measurement, through multi-cycle non-coherent accumulation, the noise is smoothed out and the position of the main peak still remains steadily pointing towards the target frequency.

**Figure 6:**
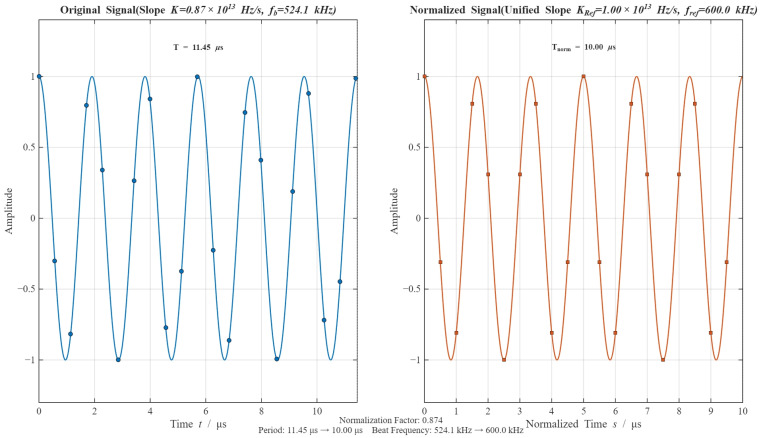
Process of NRIR algorithm.

**Figure 7:**
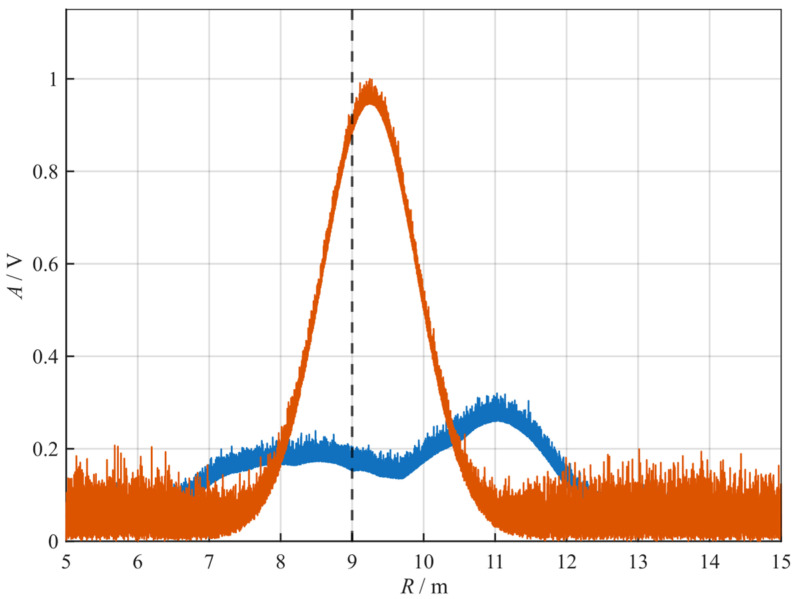
Comparison of NRIR and FFT on ranging determination. Blue curve: FFT, Orange Curve: NRIR.

As a result, the NRIR algorithm is applicable to the fuze system with short pulse width and wide bandwidth (compared to pulse width) in order to ignore the Doppler frequency shift. The algorithm flow chart is shown in Algorithm 1 and the algorithm processing procedure simulation results are presented in Figure 6 and Figure 7. The dots in Figure 6 represent the sampling and resampling data points.

It can be seen from the simulation results in Figure 7 that after resampling, the slope and beat frequency of the original beat signal are unified to $K_{\mathrm{Ref}}$ and $f_{\mathrm{Ref}}$ respectively. The spectral dispersion effect caused by slope hopping results in the FFT ranging spectral lines being scattered over a wide bandwidth, which makes it impossible to achieve effective ranging in Figure 7. After processing with the NRIR algorithm, a significant peak appears near 9 m, which verifies the effectiveness of the proposed algorithm.

**Algorithm 1** Resampling Transform Algorithm1: **Input:** Original sampling sequence $y_{n}[m]$, transmit slope $K_{n}$, reference slope $K_{\mathrm{Ref}}$
2: **Output:** Range estimate *R*3: Calculate resampling factor: $\alpha_{n}=K_{\mathrm{Ref}}/K_{n}$
4: Define new time variable: $\tilde{t}=\frac{K_{n}}{K_{\mathrm{Ref}}}t=\frac{1}{\alpha_{n}}t$
5: Calculate new time points: ${\tilde{t}}_{\mathrm{req}}=\alpha_{n}t_{k}=\alpha_{n}kT_{s}$
6: Interpolate original sequence: $y_{n}[k]=\mathrm{Interp}(y_{n}[m],\tilde{t}=\alpha_{n}kT_{s})$
7: Transformed signal: $y_{n}(\tilde{t})=\exp\left(-j2\pi K_{\mathrm{Ref}}\tau {\tilde{t}}^{\prime }\right)$
8: Apply FFT transform to $y_{n}(\tilde{t})$ to obtain peak frequency $f_{\mathrm{peak}}$
9: Calculate beat frequency: $f_{\mathrm{beat}}=K_{\mathrm{Ref}}\tau$
10: Calculate range: $R=\frac{c\cdot f_{\mathrm{peak}}}{2K_{\mathrm{Ref}}}$


### 4.3. Analysis of Algorithm Complexity

Compared with the standard FFT, the NRIR algorithm adds a resampling coordinate calculation and interpolation operation.

In resampling coordinate calculation, to calculate $t^{\prime }=\alpha_{n}t$, N multiplications are required. In interpolation operation, we use linear interpolation. Each new sampling point requires two multiplications and one addition. There is a total of 3N operations.

Assume that the number of each period sampling points is N. The standard FFT uses the Cooley–Tukey algorithm. The algorithm complexity comparison of the standard FFT and NRIR algorithm (linear interpolation) is shown in Table 1.

The additional computational load introduced by the NRIR algorithm is mainly linear O(N). For typical fuze sampling points (such as *N* = 1024), the computational burden brought by linear interpolation only increases by 8%. This is fully controllable within the processing capabilities of modern low-power FPGAs (such as Xilinx Artix-7 series). Therefore, this algorithm is highly feasible in engineering applications.

## 5. Discussion and Analysis

### 5.1. Range Resolution

Based on the physical meaning of the autocorrelation ambiguity function, when $f_{d}=0\;\mathrm{MHz}$, $\chi_{hma}$ is the range autocorrelation function [13]. Substituting into Equation (24), the range autocorrelation function of the chaotic code composite modulated signal can be expressed as

(46) $$\chi_{hma}(\tau ,0)|=\left|\sin c\left(\pi B\tau \left(1-\frac{\left|\tau \right|}{T}\right)\right)\cdot \mathrm{tri}\left(\frac{\tau }{T_{c}}\right)\right|$$

where $T_{c}$ is the symbol width; T is the modulation period; tri(⋅) is the triangular wave envelope determined by the symbol.

**Figure 8:**
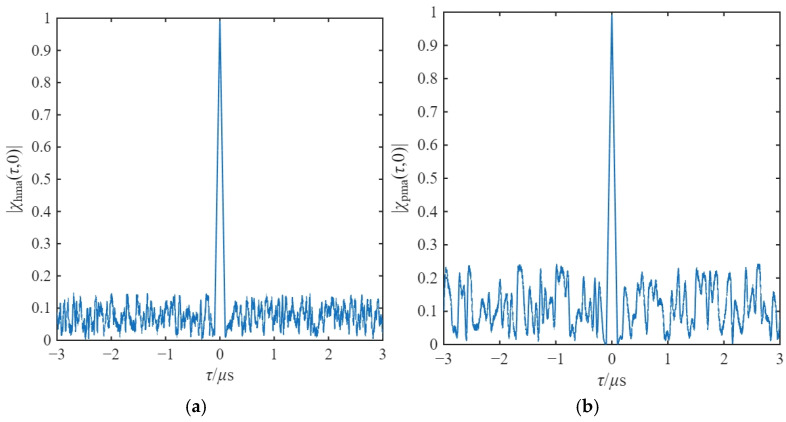
Distance auto-correlation function. (**a**) Hybrid modulated signal; (**b**) chaotic phase-modulated signal.

Its range autocorrelation function is determined by the autocorrelation performance of the chaotic code. In addition, the range autocorrelation function of the composite modulated signal is related to the modulation bandwidth of the frequency modulation component. Therefore, the main lobe of its autocorrelation function is sharper and the sidelobes are lower. The diagram of its range autocorrelation function and that of the chaotic code phase-modulated range autocorrelation function are shown in Figure 8, and the results are consistent with the above analysis.

According to the definition of range resolution, it is necessary to solve the position where |χ(τ,0)| drops from the peak value to the first zero point $\tau_{0}$. Based on R=cτ/2, it can be obtained that

(47) $$\Delta R=\left\{\begin{array}{c} \frac{c}{2B},B\geq \frac{1}{T_{c}}\\ \frac{cT_{c}}{2},B<\frac{1}{T_{c}} \end{array}\right.$$

The range resolution of the composite modulated signal is jointly determined by the symbol width and the modulation bandwidth. When $B\geq 1/T_{c}$, the range resolution of the composite modulated signal depends on the modulation bandwidth; when $B<1/T_{c}$, the range resolution of the composite modulated signal depends on the symbol period.

### 5.2. Velocity Resolution

Based on the physical meaning of the autocorrelation ambiguity function, when τ=0, $\chi_{hma}$ is the velocity autocorrelation function.

Since τ=0 leads to code alignment and thus $p_{n}(t)p_{n}^{*}(t)\equiv 1$, and the quadratic phase of the frequency modulation component is canceled out, substituting into Equation (24), the velocity autocorrelation function of the chaotic code composite modulated signal can be obtained as follows:

(48) $$\left|\chi (0,f_{d})|=\left|\frac{1}{T}\int_{0}^{NT_{p}}e^{j2\pi f_{d}t}dt\right|=|\sin c(\pi f_{d}T)\right|$$

where $T=P\cdot T_{c}$ is a complete coherent integration period.

Referring to the derivation of range resolution in Section 4.1, the first zero point $f_{d_{0}}=1/T$ is solved. The nominal Doppler resolution is half the width of the zero point [17], which is given by

(49) $$f_{d,\mathrm{Res}}=\frac{1}{PT_{c}}$$

Substituting according to the Doppler formula yields

(50) $$\Delta v=\frac{\lambda }{2PT_{c}}$$

where *P* is the number of symbols within one modulation period.

It can be seen from Equation (50) that the velocity resolution improves with the increase in symbol width or the number of symbols within each frequency modulation period.

### 5.3. Analysis of Anti-Jamming Performance

#### 5.3.1. DRFM Jamming

A Digital Radio Frequency Memory (DRFM) jammer typically intercepts the transmitted signal of a radar and retransmits it with a time delay to generate false targets. Assume that the jammer intercepts the transmitted signal $s_{tx,n}(t)$ of the *n*-th period and retransmits it after a storage delay $\tau_{J}$. The jamming signal can be expressed as

(51) $$J_{DRFM}(t)=A_{J}p_{n}(t-\tau_{J})\exp\{j\left[2\pi f_{0}(t-\tau_{J})+\pi K{\left(t-\tau_{J}\right)}^{2}\right]\}$$

The beat frequency signal obtained after mixing is given by

(52) $$y_{J}(t)=A_{J}p_{n}(t)p_{n}(t-\tau_{J})\cdot \exp\left\{-j2\pi K\tau_{J}t+\phi_{J}\right\}$$

where $\phi_{J}$ is a constant phase term independent of time.

By performing matched filtering on the beat frequency signal, assuming that the integration time is the modulation period T, the processed output is expressed as

(53) $$I_{DRFM}=\frac{A_{J}e^{j\phi_{J}}}{T}\int_{0}^{T}\left[p_{n}(t)p_{n}(t-\tau_{J})\right]e^{-j2\pi K\tau_{J}t}dt$$

In Equation (43), for the target echo, the integral term of the code product $p_{n}(t)p_{n}^{*}(t)\equiv 1$ is converted into the integral of a single-frequency signal, and the energy is coherently superimposed to form a sharp peak. For the DRFM jamming signal ($\tau_{J}\gg T_{c}$), due to the noise-like autocorrelation properties of the chaotic code, symbol misalignment causes the code product to behave as a zero-mean pseudo-random sequence. Consequently, the jamming energy is dispersed across the entire processing bandwidth *B*, precluding the formation of a coherent ranging peak [18].

According to the definition of the Peak-to-Autocorrelation Sidelobe Ratio (PASR), the PASR of the composite modulated signal can be expressed as

(54) $$\begin{array}{c} \Delta_{PASR}=\underset{\tau >T_{c}}{\sup}\left[\frac{|\chi_{hma}(\tau ,0){|}^{2}}{|\chi_{hma}(0,0){|}^{2}}\right]=\underset{\tau >T_{c}}{\max}\{|\sin c\left[\pi B\tau \left(1-\frac{\left|\tau \right|}{T}\right)\right]|\cdot \\ \frac{1}{P}|\sum_{k=0}^{P-1-m}c_{k}c_{k+m}e^{-j\theta_{k.m}}+\sum_{k=P-m}^{P-1}c_{k}c_{k+m-p}e^{-j\theta_{k,m}^{\prime }}|\} \end{array}$$

where $e^{-j\theta_{k,m}}$ is the residual phase term generated by the coupling between the linear frequency modulation component and the time delay.

When $\tau >T_{c}$, the modulus value of the sinc function must be less than or equal to 1 and decays rapidly with the increase in τ, from which it can be derived that

(55) $$\sqrt{\Delta_{PASR}}<\underset{m\neq 0}{\max}\left\{\frac{1}{P}\left|\sum_{k=0}^{P-1-m}c_{k}c_{k+m}\right|\right\}$$

For a chaotic binary phase-coded sequence with length *P*, the jamming suppression coefficient of the system against DRFM jamming is given by

(56) $${\mathrm{JSC}}_{DRFM}=\frac{1}{\Delta_{PASR}}$$

The introduction of long-period chaotic code modulation (e.g., when *P* = 1024, the sidelobe can be reduced to below −30 dB) can significantly decrease the range sidelobe and enhance the anti-jamming capability.

#### 5.3.2. Sweep-Frequency Jamming

For sweep-frequency jamming, its signal form $J_{\mathrm{sw}}(t)$ only contains the linear frequency modulation characteristic, without the chaotic code modulation $p_{n}(t)$ in the local reference signal. The processing procedure is equivalent to calculating the cross-correlation function between the local reference code $p_{n}(t)$ and the non-coded signal.

According to the characteristics of the chaotic sequence, its cross-correlation sidelobe level is generally not higher than the autocorrelation sidelobe level:

(57) $$\sup{\left|\chi_{hma}(\tau )\right|}^{2}\leq \underset{\tau \neq 0}{\sup}{\left|\chi_{hmc}(\tau )\right|}^{2}$$

Moreover, due to the absence of code modulation, the energy is spread over the entire time–bandwidth product, and the jamming suppression coefficient of the system against sweep-frequency jamming can be obtained as

(58) $${\mathrm{JSC}}_{sw}=B\cdot T$$

This equation indicates that the anti-sweep-frequency jamming capability of the composite system fuze depends on the jamming power, and it has a certain degree of anti-sweep-frequency jamming capability. However, when the power of the sweep-frequency jamming is sufficiently high, it can interfere with the composite system fuze [19].

In summary, the composite modulated signal has excellent correlation characteristics. It exhibits outstanding performance in reducing the probabilities of false alarm and missed alarm, as well as resisting active deceptive jamming, thus possessing superior anti-jamming.

## 6. Simulation and Experimental Verification

**Figure 9:**
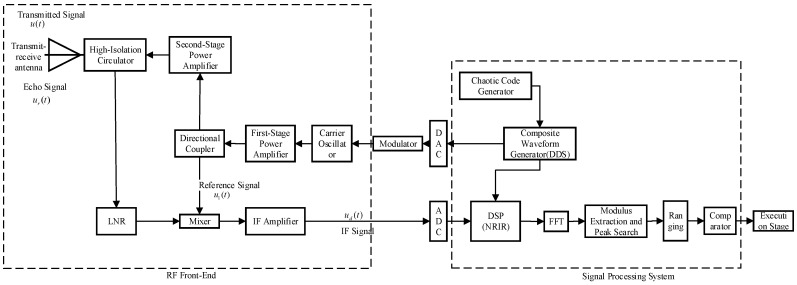
Block diagram of hybrid modulation fuze.

In this section, the anti-jamming performance of the chaotic code frequency-phase composite modulated fuze proposed in this paper is verified through Simulink simulation tests and anechoic chamber actual measurement. The block diagram of the composite modulated fuze is shown in Figure 9 and the parameters of Simulink are shown in Table 2.

The predetermined burst distance is set to 9 m. Accordingly, a reference delay $\tau_{\mathrm{Ref}}\approx 60\;\mathrm{ns}$ is applied to the receiver’s local oscillator path during the simulation to align the chaotic codes at the burst point.

### 6.1. Simulation Experimental Results

**Figure 10:**
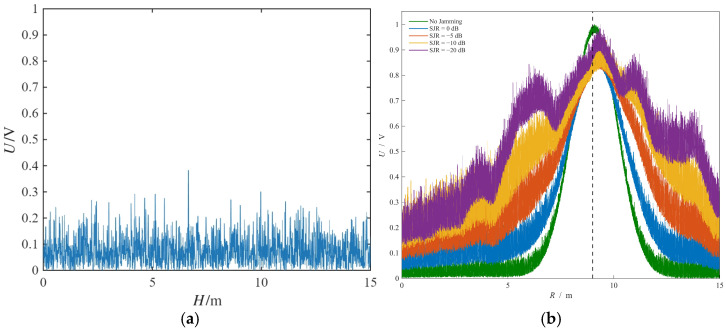
Anti-DRFM jamming effect diagram of hybrid fuze: (**a**) detection output under target-free DRFM jamming; (**b**) range determination effect diagram under different SJRs.

The detection output and ranging images under the conditions of no target and DRFM jamming with different signal-to-jamming ratios (SJR) are shown in Figure 10a.

In the case of no target and jamming presence, since the code product behaves as a pseudo-random sequence with a mean value of 0, the jamming signal energy is spread out, and the detection output energy is approximately equivalent to white noise. Under the condition of no jamming, the simulation results show that its 3 dB bandwidth is about 2.5 m. The range resolution is consistent with the theoretical analysis in Equation (37) of Section 4.1.

**Figure 11:**
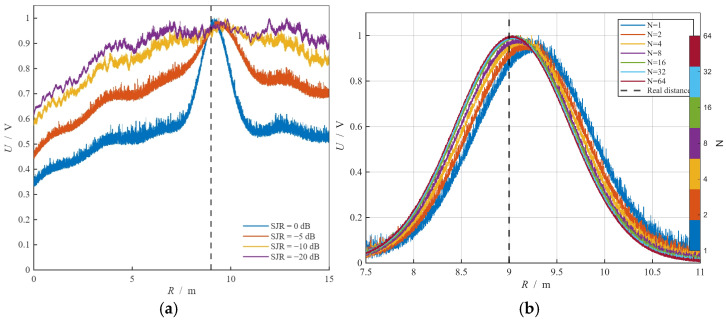
Anti-sweep-frequency jamming effect diagram of hybrid fuze and ranging effect of *N*: (**a**) anti-sweeping jamming effect diagram of hybrid fuze; (**b**) relationship between NRIR and value of *N*.

As shown in Figure 10b, the composite modulation system demonstrates robust anti-jamming performance against DRFM interference for SJRs ranging from −10 dB to 0 dB. However, at an SJR of −20 dB, the sidelobe energy becomes nearly equivalent to the main-lobe peak. This degradation occurs because the significantly higher jamming power, despite being attenuated proportionally to the symbol length according to Equation (48), results in residual energy that is comparable to or exceeds the target signal, thereby successfully disrupting the fuze. To further investigate the anti-jamming performance of the composite system fuze, sweep-frequency jamming simulation experiments were carried out. The ranging effect diagrams under different signal-to-jamming ratios (SJR) are shown in Figure 11.

The composite modulation system demonstrates robust anti-jamming performance for SJRs ranging from 0 dB to −5 dB. At an SJR of −10 dB, although the sweep-frequency jamming energy is dispersed across the entire frequency band, it is simultaneously amplified by the multi-period accumulation inherent to the NRIR algorithm. Consequently, the jamming intensity becomes comparable to the target’s ranging peak. When the SJR drops to −20 dB, the fuze completely loses its ranging functionality, a result consistent with the theoretical analysis presented in Section 4.3.

The simulation results of the ranging effect for different numbers of accumulation cycles *N* are shown in Figure 11b.

As the number of periodic accumulations increases, the ranging accuracy is gradually improved and the amplitude is also increased gradually. However, due to the large computational complexity of the NRIR algorithm, an excessive number of periodic accumulations will seriously affect the real-time performance of ranging. Therefore, the number of periodic processing cycles is set as *N* = 8.

The proposed composite fuze system demonstrates robust anti-jamming performance against both DRFM and sweep-frequency interference. Integrated with the NRIR algorithm, the system achieves high-precision ranging within hostile electronic warfare environments. To evaluate its effectiveness, the system is compared with several chaotic-based signal designs and anti-jamming algorithms, including chaotic code phase modulation, the ICHD algorithm [19], sliding multi-cycle FFT processing [20], and wavelet-transform-based signal reconstruction [21]. The Peak-to-Sidelobe Ratio (PSLR) serves as the primary performance metric for these comparative experiments, which were conducted at a signal-to-jamming ratio (SJR) of −5 dB. The results, summarized in Table 3, indicate that the proposed method yields significantly higher PSLR values against both DRFM and sweep-frequency jamming, thereby confirming its superior performance.

### 6.2. Test Results in Anechoic Chamber

**Figure 12:**
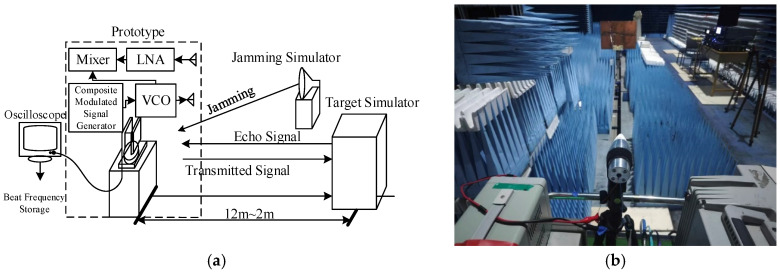
Experimental scene diagram: (**a**) anechoic chamber experiment scene schematic diagram; (**b**) head-on encounter scene schematic diagram.

**Figure 13:**
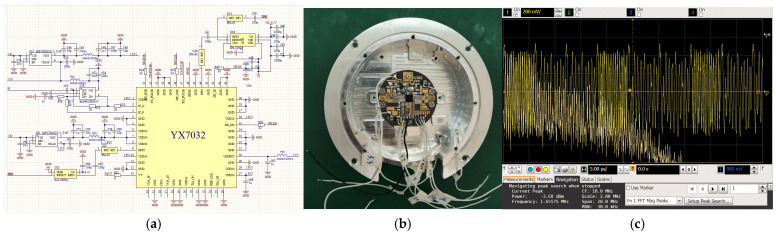
Prototype photos: (**a**) prototype schematic diagram; (**b**) physical prototype diagram; (**c**) prototype beat signal waveform under jam-free conditions.

The ranging performance and anti-active deceptive jamming performance of the composite modulated fuze were tested in a microwave anechoic chamber, and the diagrams of the test scenarios are shown in Figure 12. The schematic diagram and physical prototype diagram are shown in Figure 13.

The bandwidth of the composite modulated fuze was set to 30 MHz, and the remaining parameters were kept consistent with those listed in Table 2. The experimental procedure is described as follows: a metal flat plate with an area of 1 m^2^ was placed at a miss distance of 12 m from the fuze prototype, and then moved towards the prototype at a velocity of 1 m/s. Under the jamming environment, the detection output of the oscilloscope and the detonation signal were observed and recorded.

The beat frequency signal output by the prototype under normal operating conditions is shown in Figure 13c. It can be observed that the modulation frequency varies between two values, 100 kHz and 200 kHz.

**Figure 14:**
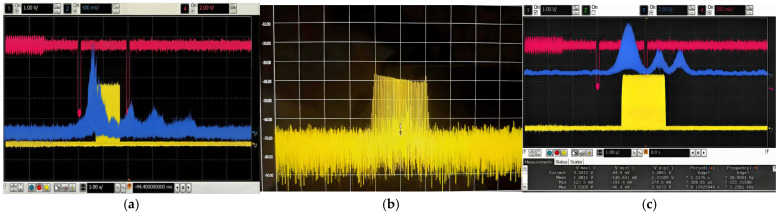
Waveform diagrams of anti-jamming performance test: (**a**) ranging waveform under DRFM jamming; (**b**) sweep-frequency jamming spectrum diagram (central frequency: 3 GHz, bandwidth: 20 MHz/div); (**c**) ranging waveform under sweep-frequency jamming.

To calibrate the distance, two phototubes were placed at the detonation point with a tolerance of 9 m±1 m. The detection output and detonation signal of the prototype under DRFM jamming conditions are shown in Figure 14a.

Following processing via the NRIR algorithm, the detection envelope peak is observed at approximately 8.3 m, fulfilling the specified ranging requirements. Moreover, due to the orthogonality of the code elements, the sidelobe energy associated with DRFM jamming is significantly suppressed. This demonstrates that the proposed fuze system possesses robust anti-DRFM jamming capabilities.

For sweep-frequency jamming, the anti-jamming experimental procedure is identical to that of the DRFM jamming experiment. The spectrum image of the jamming signal, together with the detection output and detonation signal under sweep-frequency jamming, is shown in Figure 14b,c.

Under sweep-frequency jamming, the resulting sidelobes are significantly higher than those observed under equivalent DRFM jamming conditions. This indicates that the composite fuze system is relatively more susceptible to sweep-frequency interference. Nevertheless, the main lobe of the detection envelope remains clearly discernible, with the measured detonation distance reaching approximately 8.6 m, which satisfies the operational ranging requirements. These experimental results demonstrate that the composite fuze system maintains robust anti-jamming performance against both DRFM and sweep-frequency jamming.

**Figure 15:**
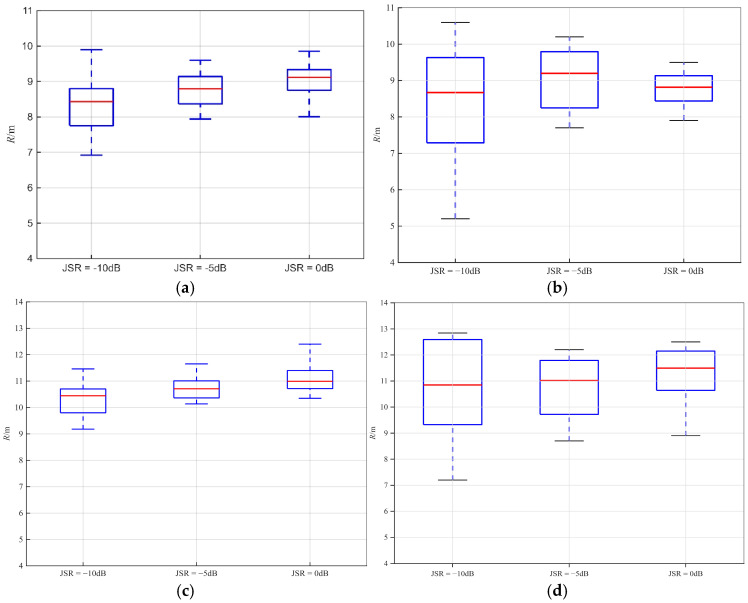
NRIR algorithm and standard FFT ranging result box plot under jamming: (**a**) DRFM jamming ranging result box plot of NRIR; (**b**) sweep-frequency jamming ranging result box plot of NRIR; (**c**) DRFM jamming ranging result box plot of FFT; (**d**) sweep-frequency jamming ranging result box plot of FFT.

The anti-jamming performance tests were conducted 20 times each under different signal-to-jamming ratios (SJR). And a comparative experiment was conducted using the NRIR algorithm and the standard FFT to evaluate their performance. The ranging boxplots of the NRIR algorithm and FFT were plotted accordingly under DRFM jamming and sweep-frequency jamming in Figure 15.

For the NRIR algorithm, at 0 dB SJR, the ranging accuracy reaches ± 0.1 m with a small data dispersion. At −10 dB SJR, the anti-DRFM jamming performance is significantly superior to the anti-sweep-frequency jamming performance, and the data dispersion is larger compared with the ranging results at 0 dB SJR. Nevertheless, the average ranging accuracy under both types of jamming still maintains ± 0.5 m, which satisfies the specified requirements.

For the standard FFT algorithm, the mean ranging values remain approximately 11 m across the SJR range of 0 dB to −10 dB. Against DRFM jamming, the proposed composite modulation system exhibits robust anti-interference performance, characterized by a narrow distribution of ranging estimates. However, due to the spectral dispersion effect, the ranging results remain near 11 m regardless of interference intensity, failing to satisfy precision requirements. Under sweep-frequency jamming, as the interference intensity increases, the variance of the ranging results broadens—with maximum values reaching approximately 13 m—while the mean remains near 11 m. Compared to the standard FFT, the NRIR algorithm improves ranging accuracy by approximately 1.7 m under DRFM jamming and 2.3 m under sweep-frequency jamming, thereby validating the effectiveness of the proposed method.

## 7. Discussion

This study proposed a chaotic frequency–phase hybrid modulation waveform combined with a Normalized Ratio-based Inverse Resampling (NRIR) algorithm to enhance the survivability of FMCW fuzes. The results demonstrate that this architecture effectively balances high ranging accuracy with robust anti-jamming capabilities.

- Interpretation of Anti-Jamming Mechanism

The simulation and anechoic chamber results (Figure 11) confirm that the hybrid modulation significantly disrupts the coherence of jamming signals. By introducing the Logistic chaotic map into both the frequency slope and phase coding, the waveform exhibits a “thumbtack-like” ambiguity function. Unlike traditional linear FM signals, the proposed waveform ensures that when a DRFM jammer retransmits the intercepted signal, the mismatch in chaotic sequences spreads the jamming energy across the time–frequency plane. This effectively transforms coherent deception jamming into background noise, maintaining a high SJR gain after processing.

- Efficacy of the NRIR Algorithm

A critical contribution of this work is the validation of the NRIR algorithm in solving the spectral dispersion problem inherent in variable-slope modulation. Traditional FFT processing fails to integrate energy from signals with time-varying slopes (as shown in Figure 8). The NRIR algorithm successfully maps the non-stationary, parameter-agile signal into a logically stationary signal through time-domain resampling. This enables coherent integration over multiple periods. Experimental data indicates that the system maintains a ranging accuracy of ±0.1 m even under a low SJR of −10 dB, proving its robustness in severe electromagnetic environments.

- 2D-FFT Progress

The NRIR algorithm operates strictly in the fast-time domain. By resampling the receive signal $y_{n}(t)$ with the factor $\alpha_{n}=K_{\mathrm{Ref}}/K_{n}$, we align the beat frequencies of all pulses to a unified reference slope $K_{\mathrm{Ref}}$. This ensures coherent focusing in the range dimension. However, NRIR does not alter the phase modulation encoded in the slow-time domain. The signal phase in the *n*-th period still contains the chaotic code term $c_{k,n}$.

If we perform a standard FFT along the slow-time dimension immediately after NRIR, the energy will not focus. This is because the chaotic phase code $c_{k,n}$ acts as a random phase shuffle from pulse to pulse. The signal in the second domain behaves like spread-spectrum noise, which is exactly the desired feature for anti-jamming/LPI (Low Probability of Intercept).

To extract the target velocity, we must perform code stripping. Since the fuze system knows the chaotic sequence $c_{k,n}$ used for transmission, we multiply the NRIR-processed signal by the conjugate of the code sequence. This restores the phase coherence across pulses:

(59) $${\mathrm{Signal}}_{\mathrm{after}\_\mathrm{strip}}={\mathrm{Signal}}_{\mathrm{NRIR}}\times c_{k,n}^{*}\approx A\cdot e^{j2\pi f_{d}nT_{PRI}}$$

**Figure 16:**
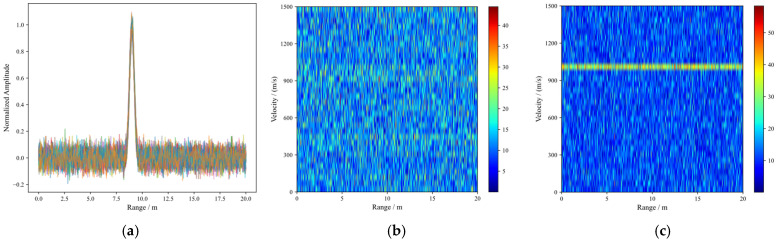
Schematic diagram of the 2D-FFT processing procedure: (**a**) range only; (**b**) 2D-FFT without stripping; (**c**) 2D-FFT with stripping.

Only after this step does the 2D-FFT yield a sharp peak in the velocity dimension. The simulation result is shown in Figure 16.

Figure 16a demonstrates that after NRIR processing, the peak positions of different pulses in the range dimension coincide, confirming that NRIR effectively resolves range defocusing issues.

Figure 16b: Despite successful range alignment, the Doppler spectrum displays significant energy dispersion along the velocity axis, with no discernible target peak. This indicates that applying a second-dimensional FFT to unprocessed, code-modulated data indeed yields an incoherent and unresolved spectral pattern.

Figure 16c: Upon application of the complex conjugate multiplication, a narrow, high-amplitude peak emerges clearly in the Doppler domain. This result substantiates the necessity of a two-stage signal processing strategy—NRIR for range compression and code stripping for velocity resolution—both being indispensable for complete target detection.

Since the main focus of this paper’s fuze system is on the ranging performance, the information related to the speed dimension has not been processed. To further enhance the anti-jamming performance of the fuze, subsequent steps can involve extracting the speed information to improve the anti-jamming capability [22].

- Limitations and Future Work

Despite these advantages, the implementation of the NRIR algorithm introduces higher computational complexity compared to standard FFT processing with higher-order interpolation. Therefore we employ linear interpolation in this paper, imposing stricter requirements on the real-time processing capabilities of the fuze processor [23]. Additionally, the wideband nature of the chaotic signal requires high linearity from the Voltage Controlled Oscillator (VCO) to avoid self-induced distortion. Future research will focus on optimizing the algorithm for FPGA-based parallel processing to reduce latency and evaluating the system’s performance in complex clutter environments involving multi-target scenarios.

## 8. Conclusions

Addressing the weak survivability of existing FMCW fuzes against sweep and DRFM jamming, this paper proposed a chaotic code slope and phase composite modulation fuze design and NRIR ranging algorithm.

- By introducing Logistic chaotic mapping, the transmitted waveform’s frequency slope and phase code possess unpredictable random jumping characteristics, providing good autocorrelation and resolution.
- The mathematical analysis of failure mechanisms proves that the composite modulation converts coherent jamming into wideband noise, which is effectively filtered by the system.
- The NRIR algorithm successfully maps physically parameter-agile signals to logically constant-parameter signals via time-domain resampling, achieving high-precision ranging under low SJR and mitigating FFT spectral dispersion.
- The system exhibits robust anti-jamming performance against both DRFM and sweep-frequency jamming, demonstrating strong robustness for complex battlefield environments.

## Figures and Tables

**Table 1:** Comparison of algorithm complexity.

Algorithm Step	FFT	NRIR (Linear Interpolation)
Coordinate mapping	0	N
Resampling/Interpolation	0	3N
FFT	$5N\log_{2}N$	$5N\log_{2}N$
Total computational load (*N* = 1024)	51,200 FLOPs	55,296 FLOPs
Relative increase amount	-	+8%

**Table 2:** Simulink simulation parameter settings.

Parameter	Value
Symbol Width	20 ns
Carrier Frequency	3 GHz
Modulation Frequency	100, 200 kHz
Modulation Bandwidth	30 MHz
Signal-to-Jamming Ratio (JSR)	−20–0 dB
Sweep Frequency	24.8–5.2 GHz
DRFM Forwarding Delay	20–100 ns
Relative Velocity	1000 m/s
Simulation Distance	15 m–0 m

**Table 3:** Comparison of PSLR for different anti-jamming methods.

Method	DRFM Jamming PSLR (dB)	Sweep-Frequency Jamming PSLR (dB)
Chaotic Phase Modulation	6.4	3.2
ICHD Algorithm	6.7	4.1
Sliding Multi-period FFT	2.1	6.5
Wavelet Reconstruction	5.6	4.4
Proposed Method	7.9	5.7

## Data Availability

Dataset available on request from the authors.

## Abbreviations

The following abbreviations are used in this manuscript:

LNA	Low Noise Amplifier
VCO	Voltage-Controlled Oscillator
NRIR	Normalized Rate-Invariant Ranging algorithm
FMCW	Frequency-Modulated Continuous Wave
UWB	Ultra-Wideband
DRFM	Digital Radio Frequency Memory
SJR	Signal to Jamming ratio
FFT	Fast Fourier Transform
